# Novel application of metal-clip traction-assisted endoscopic intermuscular dissection for a rare calcifying fibrous tumor

**DOI:** 10.1055/a-2772-6004

**Published:** 2026-01-29

**Authors:** Suhuan Liao, Silin Huang, Qi Sun, Miao He, Yin Xiao

**Affiliations:** 1 Department of Gastroenterology, South China Hospital, Medical School, Shenzhen University, Shenzhen, China; 2 Department of Pathology, Nanjing Drum Tower Hospital, Affiliated Hospital of Medical School, Nanjing University, Nanjing, China

**Fig. 1:**
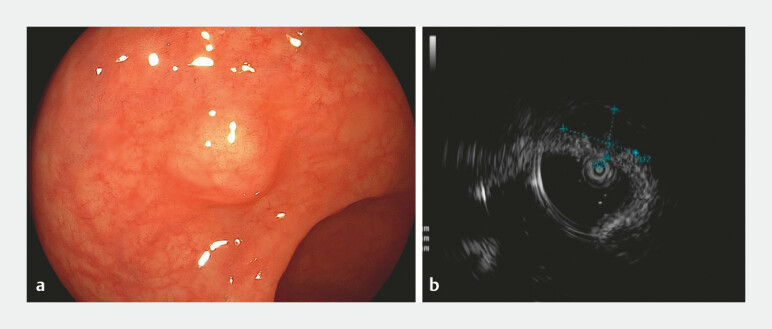
**a** Colonoscopy revealed a rectal subepithelial lesion measuring approximately 12 mm in diameter. **b** Endoscopic ultrasonography revealed a hypoechoic lesion originating from the submucosa and was closely related to the muscularis propria, exhibiting homogeneous echotexture.

**Fig. 2:**
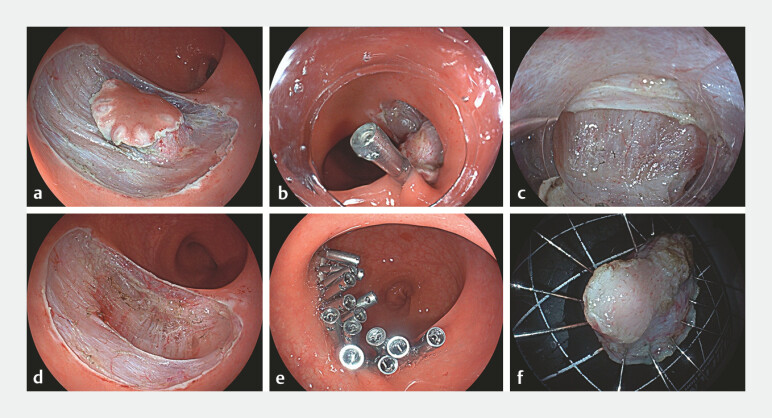
**a** The muscularis propria was exposed by mucosal incision and submucosal dissection. **b** A metal-clip traction was used. **c** With traction, the intermuscular space was adequately exposed. **d** Postoperative trauma. **e** Suture the defection with metal clips. **f** Resected tumor.

**Fig. 3:**
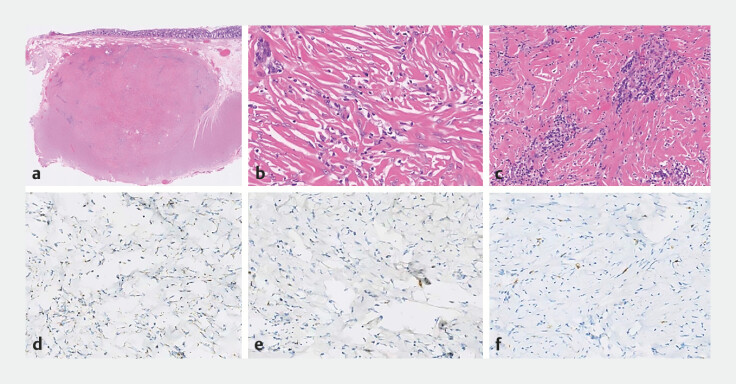
Histological findings. **a** Low-power field, HE staining: the submucosal tumor was directly adjacent to the muscularis propria, with the circular muscle layer visible beneath it. **b, c** High-power field, HE staining: a hypocellular tumor composed of collagenous fibrous tissue with scattered inflammatory cell infiltrates. **d** High-power field, positive with SDHB. **e, f** High-power field, negative for DOG-1 and CD-117.

A 34-year-old man was admitted for endoscopic resection of a 12-mm rectal subepithelial lesion (SEL; Fig. 1 **a** ). Preoperative abdominal magnetic resonance imaging showed no evidence of lymphadenopathy or distant metastasis. Endoscopic ultrasonography (EUS) revealed a tumor that was predominantly located in the submucosa and was closely related to the muscularis propria, exhibiting homogeneous hypoechoic ( Fig. 1 **b** ). In order to ensure the complete removal of the lesion, endoscopic intermuscular dissection (EID) was performed ( Video 1 ). We used a EG-601WR gastroscope (Fujifilm, Tokyo, Japan) and resected the tumor using a dual knife. The primary challenge in EID involves the dissection of the intermuscular plane. To enhance visualization of the surgical field, we employed an ST hood and a metal-clip traction technique, with the intermuscular dissection being carried out in the Endocut I mode (effect 2, duration 3, and interval 3) of the VIO200-D electrosurgical system. The procedure was continued under this approach until complete tumor resection was achieved ( Fig. 2 **a–f** ). The total procedure time was 50 minutes. Histopathological examination showed a hypocellular tumor composed of collagenous fibrous tissue with scattered inflammatory infiltrates. Uniform spindle-shaped tumor cells were dispersed among dense collagen bundles. Immunohistochemistry was positive for SDHB, with a Ki67 index of 3%, and negative for CD117, CD34, DOG-1, and S-100, consistent with a calcifying fibrous tumor (CFT; Fig. 3 **a–f** ).

Resection of a rare calcifying fibrous tumor using endoscopic intermuscular dissection and metal-clip traction.Video 1

The CFT is a rare benign soft tissue lesion of unknown etiology and exceedingly rare in the rectum, with only isolated cases reported in the literature 1 2 . Definitive diagnosis depends critically on postoperative histopathological assessment and exclusion of other entities via immunohistochemical studies. EID has been primarily reported for the treatment of rectal neuroendocrine tumors and deeply invasive rectal cancers 3 4 5 . EID can also be a treatment option for diagnostically challenging rectal SELs, particularly when EUS suggests the lesion located in the deep submucosal layer, closely associated with the muscularis propria.

Endoscopy_UCTN_Code_TTT_1AQ_2AD_3AD
